# Dystocia in a Captive Reared Agouti (*Dasyprocta leporina*) in Trinidad and Tobago, West Indies

**DOI:** 10.3390/vetsci7010030

**Published:** 2020-03-04

**Authors:** Kegan Romelle Jones, Kavita Ranjeeta Lall, Gary Wayne Garcia

**Affiliations:** 1 Department of Basic Veterinary Sciences (DBVS), School of Veterinary Medicine (SVM), Faculty of Medical Sciences, The University of the West Indies (UWI), Mt. Hope, Trinidad and Tobago; 2 The Open Tropical Forage-Animal Production Laboratory (OTF-APL), Department of Food Production (DFP), Faculty of Food and Agriculture (FFA), The University of the West Indies (UWI), St. Augustine, Trinidad and Tobago; k_lee_24@yahoo.com (K.R.L.); prof.gary.garcia@gmail.com (G.W.G.)

**Keywords:** Neo-tropics, uterine inertia, Caribbean, South America, reproductive diseases, domestic livestock

## Abstract

Dystocia is a complication that occurs at parturition either due to foetal or maternal factors. This condition has been well studies in domesticated species. However, there is very little information on dystocia in the agouti (*Dasyprocta leporina*). The agouti is utilized for its meat in South America and the Caribbean. More recently, farming of these animals intensively is being practiced in the Neo-tropics. This case report attempted to provide some insight into dystocia in the agouti which has been rarely reported in animals in captivity. A female agouti weighing approximately 3 kg (kg), which was in the last stage of pregnancy, was found dead in its cage. The vulva of the animal had the hind-limbs of the offspring protruding. Upon necropsy the animal had little fat reserves and had two foetuses in the right horn of the uterus. The feet of on offspring were dislocated and exposed at the level of the vulva. Each foetus weighed approximately 200 g. The foetuses were well formed with fur, teeth and eyes. The placenta was attached to each of the foetuses. The pathological findings suggested that dystocia resulted in secondary uterine inertia, which was the cause of death of the adult female agouti. To prevent the recurrence of this situation the gestation should be staged (timed) using ultrasonography. Animals which are in their third stage of gestation should be monitored using cameras or with personnel at the facility to assist agoutis which are having difficulties at parturition.

## 1. Introduction

The agouti (*D. leporina*) is a rodent that belongs to the hysricomorphic group of rodents. It is one of the most hunted species in the Caribbean and South America [1]. The use of this mini-livestock is a major source of meat protein for rural communities in the Neo-tropics [2]. The exploitation of these animals in the wild can lead to decreasing numbers in the wild population. However, if the agouti is domesticated and reared in intensively, animals can be re-introduced into the natural environment as a conservation strategy [1].

Several articles have been published on the male anatomy of the agouti. The micro- and macro-anatomy of the male reproductive tract [3,4], the electro-ejaculates of the male agouti has been analyzed and these ejaculates have been extended and stored [4,5,6,7,8]. Finally, the stages of erection in the male agouti have been investigated [9]. The age of weaning of this precocious rodent under intensive production has been reported to be one week [10]. The female reproductive system has also been studied and will be discussed in this paper.

The nutrition and feeding practices of agouti have been documented on several occasions. The digestive tract of the agouti is similar to that of the rabbits. The agouti possessed a large caecum and the small intestine was reported to be longer in comparison to that of the rabbit [11]. Some authors have stated that the animal is a frugivore with a large majority of its diet consisting of fruit and nuts [12,13]. Researchers have also found that the animal is a grubivore, consuming insects that were present in the seed of some nuts [14]. Dookie et al. [15] reported a feed particle size preference of 12.7 mm × 25.4 mm. Lall et al. [16] reviewed the diets of these animals in captivity and in the wild. It was summarised that the agouti was frugivorous, herbivorous and opportunistic in its feeding behaviour. In captivity the consumption of animal matter has been documented with the agouti consuming carrion, chicken eggs and dead chicken [17,18].

The majority of diseases highlighted have been related to some infectious agent. Lall et al. [19] reviewed pathogenic diseases present in the agouti and found that few animals showed clinical signs of diseases. This made the agouti a major reservoir of diseases for domesticated animals. Additionally, animals found in the neotropics were grouped into three categories. (1) Domesticated animals that were introduced into the neo-tropics which were: cattle, sheep, goats, chickens, pigs and horses. (2) Domesticated animals that originated from the neo-tropics (South American camelids, chinchillas, ducks, turkeys and guinea pigs). (3) Non-domesticated animals that originated from the neotropics (agouti, lappe, capybara, red brocket deer, collared peccary and manicou) [20,21,22].

Investigation on the gastrointestinal parasites common to the agouti reared in captivity and the wild have been documented [23,24,25,26,27,28]. Blood parasites have been found in wild agouti populations. Organisms identified included *Babesia*, *Leishmania* and *Trypanosoma cruzi* [29,30]. However, in the captive reared colonies of agoutis blood parasites were not identified [31]. Few reports were given on the effects that the parasitic organisms had on the agouti. Authors who investigated the effects of the parasites on the animals found that the agouti was clinically healthy, with a body condition of 3 out of 5 [25].

Some works on the detection of subclinical illness in the agouti have been reported, discussing the haematology and serum biochemistry. Baas et al. [32] reported on blood reference values for agoutis reared in captivity. Further work by Jones et al. [31] and Jones and Garcia [33,34] recorded blood parameters for healthy agoutis reared intensively. However, little reports existed on non-infectious diseases of the agouti. However, imbalances in vitamin D have been highlighted [35,36]. There is a dearth of information on non-infectious reproductive diseases. Personal communication from the third author of this paper stated that two diseases and dystocia, which is a consecutives situation that seriously affects delivery. These were (1) pasteurellosis, (2) scabies and (3) dystocia. The objective of this report was to highlight findings of one such dystocia.

## 2. Material and Method

### 2.1. Housing of the Agouti (Dasyprocta leporina)

The maternity cages measured 0.61 m long × 0.91 m wide × 0.61 m high. Animals were separated from the colony reared on floor pens when signs of pregnancy were noted. Confirmation of pregnancy was done using physical signs such as a rounded abdomen and protruding nipples. The animal had an approximate weight of 3 kg. The female agouti was part of a colony of agoutis, which were reared intensively at the University of the West Indies Field Station, Trinidad. The colony housed one hundred agoutis, which were reared in individual cages and also in pens on concrete floors.

The diet of the animals consisted of locally available fruits such as mangoes (*Mangifera indica*), pumpkins (*Cucurbita pepo*) and fat pork (*Chrysobalanus icaco*). Chicken eggs were also fed to the animals in conjunction with commercial pelleted rabbit ration (Crude Protein 17%). Animals in the unit always had a constant supply of water for drinking and thermoregulation. The concrete floor pen consisted of breeding units with a female to male ration of 5:1. Animals which are kept at this station are under the sanction of animal welfare guideline of the University of the West Indies. In this unit there are two veterinarians which monitor and treat animals showing overt signs of disease.

### 2.2. Clinical Signs

Pregnant agoutis were observed daily for any signs of physical abnormality, which included abnormal discharges from the vulva, diarrhea and emaciation. Animals were also observed for any signs of physical trauma or wounds, which may have occurred as a result of fighting.

## 3. Results

### 3.1. Post Mortem Examination

An adult female agouti (*D. leporina*) approximately 2 years of age was found dead in a maternity cage. The hind-limbs of one offspring were protruding from the mother’s vulva. Upon presentation the dead agouti was then taken and a necropsy performed to identify any abnormalities in foetal positioning.

### 3.2. Treatment

The animal was found dead in the cage. The animal had no prior signs of illness. This situation did not allow for the agouti to be treated for its dystocia.

### 3.3. Necropsy Findings

**Figure 1:**
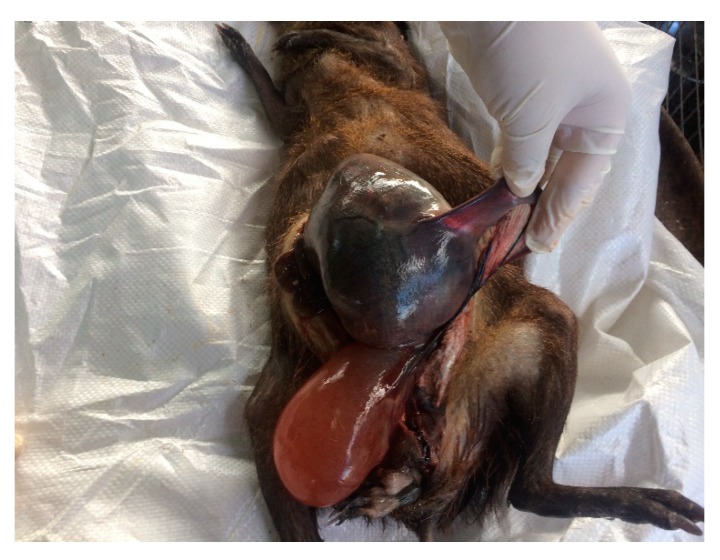
Necropsy of Adult Agouti with oversized foetus in the right uterine horn which resulted in the death of the animal.

The abdominal cavity of the agouti was opened and a right uterine horn was distended with two foetuses and the left uterine horn was empty. The urinary bladder of the animal was also distended. Figure 1 shows a part of an absolute large foetus, which resulted in the dydtocia, which consequently resulted in secondary inertia. The lack of medical attention to the secondary inertia resulted in the death of the offspring and the mother.

**Figure 2:**
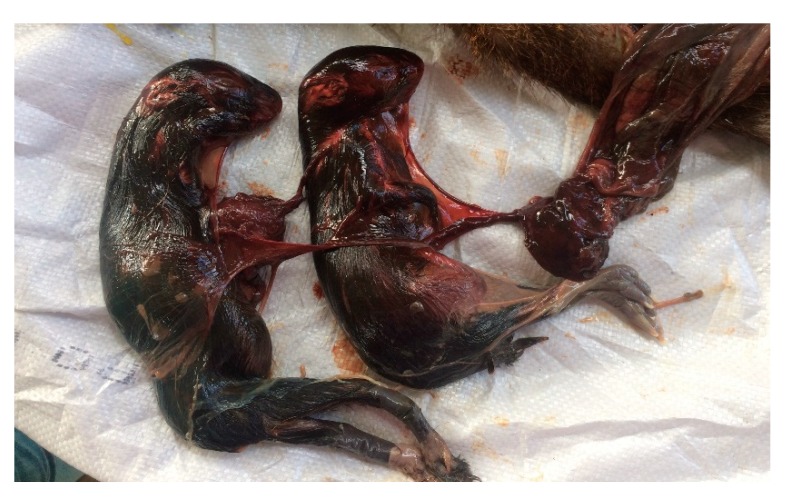
Two offspring weighing 200 g each in the uterus.

The right uterine horn was opened and two foetuses, each weighing approximately 200 g were found inside. Both foetuses were in posterior longitudinal presentation, dorsal position and in normal posture. The offspring had hair, teeth and nails with the placenta attached to each animal (Figure 2). The carcass had little subcutaneous fat and the cause of death was attributed to secondary uterine inertia due to the dystocia.

## 4. Discussion

Little information has been reported on the reproductive disorders in the agouti. Some authors have observed perinatal mortality in captive reared agoutis in Trinidad. There was one reported case in Trinidad, in which an agouti died from secondary uterine inertia due to dystocia. In that case the adult female was reported to have had six offspring. Three were delivered via the vagina but the remaining offspring were removed via caesarean section [37]. In Brazil, pathological changes in the reproductive system of captive reared breeding females were investigated. Dystocia was reported in 7.7% (n = 1) of breeding females. In that case of dystocia there was one large offspring present in a uterine horn [38]. Batista et al. [39] also reported a prevalence of 6.24% for dystocia in captive reared agoutis.

Dystocia is the inability to expel the foetus from the uterus during parturition, which occasionally occurs in domesticated animals. It can be due to maternal or foetal abnormalities. Maternal abnormalities include; small pelvic size, narrow pelvic canal and uterine inertia. Foetal causes are due to abnormal presentation, position, posture, oversized foetus, foetal death or monsters [40]. In rabbits treatment of dystocia has been done either medically or surgically. In case of any abnormal presentations forms, stimulating uterine contractility must not be performed before correction of presentation abmormalities in order to avoid uterine rupture, cervical damage or occlusion of the foetus in the birth canal. Treatment included administration of calcium borogluconate, propylene glycol, and oxytocin. Calcium and oxytocin have been reported to increase the contractility of the uterus [41]. Propylene glycol provides energy for the smooth muscles in the myometrium [41]. Surgery has to be performed when the above mentioned drugs do not result in the solution for dystocia.

In this case the animal was found dead in the morning. It is being suggested that if dystocia is encountered in the agouti in future the medical management used in the rabbit using a “dystocia cocktail” (oxytocin, propylene glycol and calcium borogluconate) could be given to the affected animal [42]. In mice with dystocia the use of these drugs are not considered as the first line of treatment. The uses of these drugs have been reported to be ineffective in treatment of dystocia in mice and authors have suggested that surgical treatment be done as the primary response to treatment [42].

In this case the cause of death was reported to be secondary uterine inertia due to dystocia. There was normal presentation, posture and position of the foetuses. However, the both foetuses were large and present in the right horn, which may have contributed to dystocia. Investigations showed elsewhere that the two uterine horns were independent and each of them was connected to separate cervix [43]. Therefore, the two large foetuses that were present in the right uterine horn would have had to pass through the right cervix, which may have contributed to the dystocia seen in this case. Further work done by Singh et al. [44] were in disagreement with Mayor et al. [43] and stated that the agouti had one cervix and the two uterine horns connected to the body of the uterus, which was connected to a common cervix. Other hystricomorphic rodents such as the capybara (*Hydrochaerus hydrochaeris*) and the lappe (*Agouti paca*/*Cuniculus paca*) had uterine horns that were independent of each other and were connected independently to two cervices [45,46].

Early observations on parturition in the agouti by Enders [46] stated that newborn animals were fully haired, had open eyes and were patterned like the adult. The animal gave birth in a squatting position having one hour intervals in delivery of offspring. Enders [47] also reported a dystocia that resulted in the death of mother and offspring. The right hind limb of the lower foetus was engaged resulting in failure to expel uterine contents. The limbs were dislocated due to trauma, which was similar to this case of dystocia seen at the University Field Station. Weir [48] also reported a case of dystocia where the foetus was reported as being oversized and was found in the right uterine horn. In this case however, the foetuses had average birth weights [47] and were found in the right horn in reports [48].

Prevention of dystocia can be done if accurate stages of gestation can be investigated. Souza et al. [49] monitored gestation age and embryonic-foetal development in the agouti. It was noted that gestational length was 103 days and, using ultrasonography the gestational sac can be observed from day nine [49]. Brown [50] stated that the gestational period for the golden-rumped agouti (*Dasyprocta aguti*) was 104 days, Pachaly et al. [51] reported a gestational period of 103 days while Clarke and Olfert [52] gave a gestational period ranging from 104–120 days. Fortes el al. [53] highlighted that at twenty-five days after mating the foetus was positioned in a “C Shape” with primitive structures. To prevent the recurrence of this situation the gestation should be staged (timed) using ultrasonography. Animals which are in their third stage of gestation should be monitored using cameras or with personnel at the facility to assist agoutis which are having difficulties at parturition. If difficulty is noticed at parturition, then the mother can be treated medically or surgically to alleviate the situation. This will avoid have avoided the deaths of the mother and fetuses which occurred in this report.

In this case the pregnant agouti carried two offspring, which was similar to the litter sizes reported in the literature. Pachaly et al. [51] reported the agouti to have had an average litter size of 2.09 and Mayor et al. [43] reported average litter sizes of 2.1. Brown-Uddenberg [54] reported average litter sizes of 1.71 and Jones and Garcia [55] found average litter sizes of 1.7.

## 5. Conclusions

In this case the cause of death was attributed to secondary uterine inertia due to dystocia. The two foetuses were found in one horn, which may have attributed to the dystocia. The absolute foetal oversize and failing medical intervention were responsible for the death of the foetus and the dam. It is generally not advised to leave periparturient animals unattended for several hours. To prevent the recurrence of this situation the gestation should be staged (timed) using ultrasonography. Animals which are in their third stage of gestation should be monitored using cameras or with personnel at the facility to assist agoutis which are having difficulties at parturition.

There has been little information reported on the reproductive disorders of the agouti. More information needs to be documented on reproductive disorders of agoutis reared in captivity. Intensification of the agouti may reveal a higher incidence of dystocia and proper treatment of such cases should be reported.
